# Testing the Level of Agreement between Two Methodological Approaches of the Rapid Upper Limb Assessment (RULA) for Occupational Health Practice—An Exemplary Application in the Field of Dentistry

**DOI:** 10.3390/bioengineering10040477

**Published:** 2023-04-15

**Authors:** Ramona Nowara, Fabian Holzgreve, Rejane Golbach, Eileen M. Wanke, Christian Maurer-Grubinger, Christina Erbe, Doerthe Brueggmann, Albert Nienhaus, David A. Groneberg, Daniela Ohlendorf

**Affiliations:** 1 Institute of Occupational Medicine, Social Medicine and Environmental Medicine, Goethe-University Frankfurt, 60596 Frankfurt am Main, Germany; 2 Institute of Biostatistics and Mathematical Modelling, University Hospital Frankfurt, 60596 Frankfurt am Main, Germany; 3 Department of Orthodontics, University Medical Center of the Johannes Gutenberg-University, 55131 Mainz, Germany; 4 Competence Centre for Epidemiology and Health Services Research for Healthcare Professionals (CVcare), University Medical Centre Hamburg-Eppendorf (UKE), 20246 Hamburg, Germany; 5 Department of Occupational Medicine, Hazardous Substances and Public Health, Institution for Statutory Accident Insurance and Prevention in the Health and Welfare Services (Berufsgenossenschaft für Gesundheitsdienst und Wohlfahrtspflege-BGW), 22089 Hamburg, Germany

**Keywords:** RULA, ergonomics, ergonomic risk assessment tools, biomechanics, dentistry, inertial motion capture, inertial motion units, Xsens, weighted Cohen’s kappa, mosaic plots

## Abstract

Background: The Rapid Upper Limb Assessment (RULA) is used for the risk assessment of workplace-related activities. Thus far, the paper and pen method (RULA-PP) has been predominantly used for this purpose. In the present study, this method was compared with an RULA evaluation based on kinematic data using inertial measurement units (RULA-IMU). The aim of this study was, on the one hand, to work out the differences between these two measurement methods and, on the other, to make recommendations for the future use of the respective method on the basis of the available findings. Methods: For this purpose, 130 (dentists + dental assistants, paired as teams) subjects from the dental profession were photographed in an initial situation of dental treatment and simultaneously recorded with the IMU system (Xsens). In order to compare both methods statistically, the median value of the difference of both methods, the weighted Cohen’s Kappa, and the agreement chart (mosaic plot) were applied. Results: In *Arm and Wrist Analysis—area A*—here were differences in risk scores; here, the median difference was 1, and the agreement in the weighted Cohen’s kappa test also remained between 0.07 and 0.16 (no agreement to poor agreement). In *area B—Neck, Trunk, and Leg Analysis*—the median difference was 0, with at least one poor agreement in the Cohen’s Kappa test of 0.23–0.39. The final score has a median of 0 and a Cohen’s Kappa value of 0.21–0.28. In the mosaic plot, it can be seen that RULA-IMU had a higher discriminatory power overall and more often reached a value of 7 than RULA-PP. Conclusion: The results indicate a systematic difference between the methods. Thus, in the RULA risk assessment, RULA-IMU is mostly one assessment point above RULA-PP. Therefore, future study results of RULA by RULA-IMU can be compared with literature results obtained by RULA-PP to further improve the risk assessment of musculoskeletal diseases.

## 1. Introduction

Ergonomics deals with the optimal adaptation of work to the human being [1]. Accordingly, the workplace must be designed, from an ergonomic point of view, in such a way that the employee is protected from injuries and illnesses, while his or her performance is ensured [2]. The assessment of potential work-related risk factors (e.g., static and/or non-physiological posture over a continuous period of time or high carrying loads) can be performed by ergonomic risk assessment tools (ERATs) [3]. They are used to identify an imbalance between the workplace conditions and the physiological capabilities of the employees [3] and appraise the applied forces, duration of physical work, and postural variability, amongst others, for risk stratification [4]. Such observation methods can cover a wide range of work situations and are also inexpensive to collect [5].

Due to the frequent use of ERATs to determine ergonomic risk, they should be applicable with a low error rate. However, 26.83% of assessments contains errors; these are partly due to a lack of knowledge or training in the proper use of the individual ERAT [6] but also to the more difficult assessment of an entire work process by an observer [7].

The S1 guideline “guideline on physical stresses on the back due to load handling and forced postures in the work process” of the Association of Scientific Medical Societies [8] recommends the Rapid Upper Limb Assessment (RULA) in Germany to determine the stress and strain on the shoulder and upper back [9] as a non-invasive measurement method. RULA is an internationally widely used and scientifically well-evaluated method [9]. However, the correlation coefficient of its inter-rater reliability of the individual steps lies within 0.54–0.72 and that of the *total score* between 0.5–0.7 in a medium range. More inconsistent is the value of the intra-rater reliability for the steps, lying between 0.27 and 0.86 and the *total score* between 0.47–0.84 [10]. Therefore, extreme body positions are often selected. In addition, the comprehensive registration of dynamic movements is missing as the human observer can only evaluate a self-selected section of the dynamic movement and, thus, cannot do justice to a complete risk assessment. However, more than 90% of the users in the studies of Diego-Mas [6,11] felt that RULA adequately reflected the current risk in relation to the workplace and working conditions and helped to initiate possible, necessary ergonomic changes to the analysed workplace [11].

Exact angle detection can be quantified in the context of a movement analysis using inertial measurement units (IMUs) [5]. IMUs are one of the direct measurement methods available and are already being employed in the automotive industry [12], offices [13], supermarket checkouts [14], and in other workplaces with manual activity [15] to measure joint angles and, thus, can help to assess more effectively and design the ergonomics of the workplace in the future. In order to be able to carry out the risk assessment independently of a subjective expert assessment, the IMUs data of the direct measurements were combined with scoring models, such as RULA [7,16,17], to objectify the assessment of the ergonomic risk of workplaces.

Therefore, the aim of the present study was to identify systematic deviations of RULA risk assessments between the conventional paper and pen method of an observer (RULA-PP) and the use of kinematic data (RULA-IMU). This comparison was made on situation recordings using the example of a dental team (consisting of a dentist and dental assistant) working in a dental treatment situation [18] as this study is part of the SOPEZ project (study for the optimization of ergonomics in the dental practice). The general aim of this project was to evaluate ergonomic risk factors in the dental setting and to provide recommendations. Further information can be found in the study protocol [18].

RULA-PP has been proven to be a valid method with only one observer required to assess work-related risk factors [19]. For the comparability of the data, the data of the IMUs were modified and integrated into the RULA steps and, among other things, certain angular ranges were defined for this purpose (see RULA modifications methodology).

The following research questions were explored:

^1.^  How does RULA-IMU relate to RULA-PP in *area A* (*Arm and Wrist Analysis*)?
^2.^  How does RULA-IMU relate to RULA-PP in *area B* (*Neck, Trunk, and Leg Analysis*)?
^3.^  How does RULA-IMU relate to RULA-PP in the RULA *total score*?
^4.^  Can recommendations be formulated for the targeted use of both methods?

## 2. Materials and Methods

### 2.1. Subjects

A total of 130 subjects (43 male/87 female) between 18 and 65 years of age took part in the study. These subjects were recruited with flyers displayed in dental offices between March 2020 and March 2021 and by word of mouth among dental students. For the measurement, the subjects were divided into pairs consisting of a dentist (D) (31 m/34 f) and a dental assistant (DA) (12 m/53 w). These pairs participated together and consisted of the D and their associated DA, just as they worked in their own practice on an everyday basis. Others consisted of dental students and their co-enrolled student peers. Primarily, only right-handers were admitted. The inclusion criteria for the participants were that they must have both theoretical and practical training in a dental profession in order to be able to adopt the required working posture in a profession-specific manner. Persons with acute injuries to the musculoskeletal system (such as herniated discs or spondylolisthesis), restrictive movement limitations with and/or malformations of the spine, spondylodeses in the past, and, also, rheumatic diseases were excluded. This study was approved by the Ethics Committee of the Medical Faculty of the Goethe University Frankfurt am Main (356/17). All participants in the study took part voluntarily, and all experiments were conducted in accordance with the relevant guidelines and regulations.

### 2.2. Measuring Systems

#### 2.2.1. Inertial Measurement Unit (IMU)

The recording of body posture was performed via the motion capture system of the company Xsens (Enschede, The Netherlands). For this purpose, 17 inertial sensors, the transmission device, and a battery were attached to a special suit. The Xsens system interpolates, among other things, 22 joints with 3 degrees of freedom (sagittal, frontal, and transverse planes) and data on the positioning and orientation of the segments. The mean measurement error lies between 1.38° and 6.69° [20]. A low frequency of 24 Hz (240 Hz are possible) was chosen for the data evaluation, as the recorded body position was maintained for 10 s (static measurement position). Since there was no floor interaction and no change in the position in the room (test persons were sitting on chairs), recording in the “no-level” function was selected according to the manufacturer’s recommendation [21]. In this recording function, the hip segment formed the reference of the individual coordination system.

#### 2.2.2. Photography: Apple iPad

An Apple iPad with a 1080P camera resolution (Cupertino, CA, USA; resolution: 1080P HD, 120 fps) was used for the photo shoots.

### 2.3. Rapid Upper Limb Assessment (RULA)

The RULA was developed in 1993 as a paper and pencil method by McAtamney et al. [9]. In order to quantify the working posture from an ergonomic point of view, an overall score was determined from 15 assessment steps, which included a risk assessment [22]. This assessment protocol was divided into two main *areas*, *A* and *B*.

In *area A—Arm and Wrist Analysis*—which covered the analysis of the shoulder, elbow, and wrist joints, points were awarded per observed angle, flexion, rotation, and lateral flexion in their three given lines of freedom. This culminated in the A score that combined the previous individual steps and, thus, provided a common representative point value for *area A*.

In *area B—Neck, Trunk, and Leg Analysis*—the deviations of the 0° position of the head, trunk, and lower extremity were analysed. These steps were summarised by the B score. In addition, in both areas for the conventional paper and pencil method, as well as in the data-driven method, one point each was added to the point value, as it has been reported that the static posture is generally maintained for a longer period than 10 s (used in the study) by practising dentists and dental assistants [23]. In those steps dealing with force and load assessment, the weight of the instruments to be handled was also assessed. Here, the score was set to 0 in the experiment, as the D’s and DA’s instruments weighed less than 2 kg. Thus, the overall result of the observed posture was scored between 1 and 7; this assessed the accompanying ergonomic risk as follows [24]:

1–2: acceptable posture;

3–4: further investigation, change may be needed;

5–6: further investigation, change soon;

7: investigate and implement change.

### 2.4. RULA Modifications

Since the original version of the RULA procedure functions on the basis of observations, there are assessment steps that are not subject to quantitative criteria. In order for the RULA procedure to work with continuous data, modifications had to be made to those steps that did not have quantitative thresholds. All modifications can be found in detail in Maurer-Grubinger et al. [25]. As an example, the following modification was made in *area A* for step 1 (upper arm score) [9]. In RULA-PP, the observer decided when there was elevation and abduction and awarded one point for each. In RULA-IMU, a point was awarded when there was an abduction angle of more than 45°. At the same time, the IMUs system calculated a + 1 in step 1 if there was an elevation of the scapulae of 5°. In both methods, supporting or leaning on the arm was awarded 0 points since, in everyday dental practice, the arm is moved freely [25].

We oriented ourselves to the work of Vignais et al. [16], who were some of the first authors that combined RULA with IMU. They defined five local scores, which we have also adopted. In addition, we used the A score and B score because they are well suited as general score for the upper extremity and the trunk position.

### 2.5. Measurement Protocol

After donning the measuring suit, the subjects performed a treatment on a phantom head within their dental treatment team (D + DA). Each subject team was measured in a typical working posture four times each, representing different arrangements of the inventory [18]. There are four workplace arrangements in dental practices used internationally, and, in these, the participants were measured. For more information on the experimental design, please refer to the study protocol of the SOPEZ project [18].

**Figure 1:**
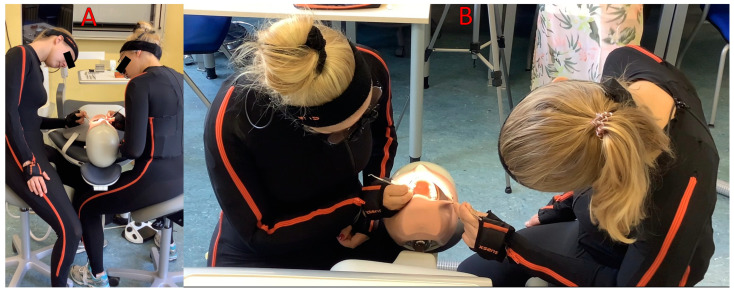
Still images for the evaluation of RULA-PP from two angles (from front (**A**) and from back (**B**)).

The side of the body that was used for the intended work was measured and included in the evaluation; in most cases, this included the right side of the body, but, for the DAs, it could also include the left (Figure 1). The starting position of each measurement was always as follows: D and DA would first examine tooth 26 (1st molar in the left quadrant of the upper jaw), which was to be treated with a swab. Both subjects were asked to maintain this position quietly for 10 s with the D holding a dental probe and the DA holding either a probe or a mirror.

All measurement sequences were photographed from two positions (front top, rear side) to ensure an optimal view for the observer’s evaluation with RULA-PP. RULA-PP was collected by one trained observer to collect RULA risk profiles.

### 2.6. Data and Statistical Analysis

Statistical data analysis was performed using Microsoft Excel 2016 (Microsoft Corporation, version 16.0, Redmond, WA, USA) and Matlab R2020a (The Mathworks Inc., Natick, MA, USA).

RULA-PP: the photographs from the two perspectives taken were viewed using Microsoft Photos (Microsoft Corporation, version 2021.21120.8011.0, Redmond, WA, USA), and the assessments according to RULA were transferred to Microsoft Excel.

RULA-IMU: all data recorded by the measuring system were checked and converted to a mat. file. The further analyses were performed with a self-written script in Matlab, version 2020a (The MathWorks, Inc., Natrick, MA, USA), the details of which are described by Maurer-Grubinger et al. [25]. First, the results of the measured RULA ranges were calculated: step 1, step 2, step 3 + 4, A score, step 9, step 10, B score, and the *total score*.

Most important for the analyses were the results of A score and B score since they summarised the results of *area A*—*Arm and Wrist Analysis*—*area B*—*Neck, Trunk, and Leg Analysis*—and the *total score*, thus representing the result of the RULA.

The Kolmogorov–Smirnov–Lilliefors test was used to test the RULA-IMU and RULA-PP data sets for normal distribution. Most of the data had not been normally distributed.

The median value of the difference of both methods (median RULA-IMU—median RULA-PP) was calculated for each RULA step, the total score, and for each setting. Therefore, in total, 32 median differences were calculated.

To highlight the differences between the two measurement methods, a method comparison was performed according to the weighted Cohen’s Kappa (k) [26] for each step and the total score and for each setting in R (Version R-4.2.1, R Core Team, Vienna, Austria) and RStudio (Version 2022.07.1 Build 554; RStudio Team, Boston, MA, USA). The Cohen’s kappa coefficient determined the degree of agreement (k) in terms of inter-rater reliability. It could be assessed as follows [27]:
k ≤ 0.1 no match0.1 < k ≤ 0.4 poor match0.4 < k ≤ 0.6 clear match0.6 < k ≤ 0.8 close match0.8 < k ≤ 1 (almost) complete match

The results of the weighted Cohen’s Kappa (k) were then visually represented as a mosaic plot, from which it could be concluded which method had the tendency towards higher scores and which score was selected most frequently in the respective RULA step.

Here, both methods were plotted against each other in a graph: In each mosaic plot shown, the *x* axis describes all point values chosen in RULA-IMU, while the *y* axis includes all results from RULA-PP.

To determine a tendency towards a particular rating for RULA-IMU, the width of the columns can be compared. A wider column for a particular rating indicates a higher proportion of assessments with the same rating. Within a column, the height of the rectangles can be compared to determine a tendency towards a particular rating for RULA-PP. Thus, the length of the rectangle on the axis implies the frequency of the score chosen: the longer the rectangle, the more often the score was used.

The extent of the difference between the two methods can be seen in the colour gradation of the squares:
red:Difference from 0orange:Difference from 1green yellow:Difference from 2green:Difference from 3turquoise:Difference from 4blue:Difference from 5lilac:Difference from 6violet:Difference from 7

To interpret the comparability of the two methods, the red squares, which indicated a difference of zero between the two methods, must be considered in relation to the 45° diagonal: in the case of perfect agreement between the two methods, the red rectangles would completely fill each column. The smaller the red rectangles, the smaller the proportion of matching ratings of both methods. The further the rectangles deviated from the diagonal, the greater the distortion between the two methods. Red rectangles above the diagonal line indicated a lower ranking of RULA-PP compared to RULA-IMU, while red rectangles below the diagonal indicated a higher ranking.

The photos and mosaic plots were further processed in FotoFiltre 7 (PhotoFiltre Studio, version 11.3.0, Antonio Da Cruz, Houilles, France).

## 3. Results

In this section, the RULA steps of both methods are directly compared with each other, e.g., step 1 of RULA-PP with step 1 of RULA-IMU.

In *area A—Arm and Wrist Analysis*—the median of the differences is one in almost all cases, after which RULA-IMU has higher scores than RULA-PP. In contrast, the median in all calculated individual steps is 0 for *area B—Neck, Trunk, and Leg Analysis*. The median of the differences of the total point value reflects the differences of the median in *areas A* and *B*, in which it assumes 0 in the minimum and 1 in the maximum (Table 1).

The weighted Cohen’s Kappa (k) reaches only a weak agreement (k ≤ 0.4) in each of the RULA steps. The values range from k = 0.01 in step 3 + 4 to k = 0.33 in B score. The *total score* has a kappa coefficient of 0.21 and indicates a weak agreement between the two methods. A clear difference is also evident in the steps that summarise their respective domains, as the score of A score, with a k of −0.07–0.08, turned out to be more different than that of B score, with a k of 0.31–0.34.

The interquartile range (IQR) varies between the values of 0 and 2, depending on the step and the step. In the overall view, the median is between 0 and 1 in *area A* and between 0 and 2 in *area B*. In the *total score*, the interquartile range is between 0 and 1 (Table 1).

Min. k: lowest weighted Cohen’s Kappa value for all four settings; max. k: highest weighted Cohen’s Kappa value for all four settings; min. median: lowest median of the difference of the methods for all four4 settings; max. median: highest median of the difference of the methods for all four settings; and IQR: interquartile range.

**Figure 2:**
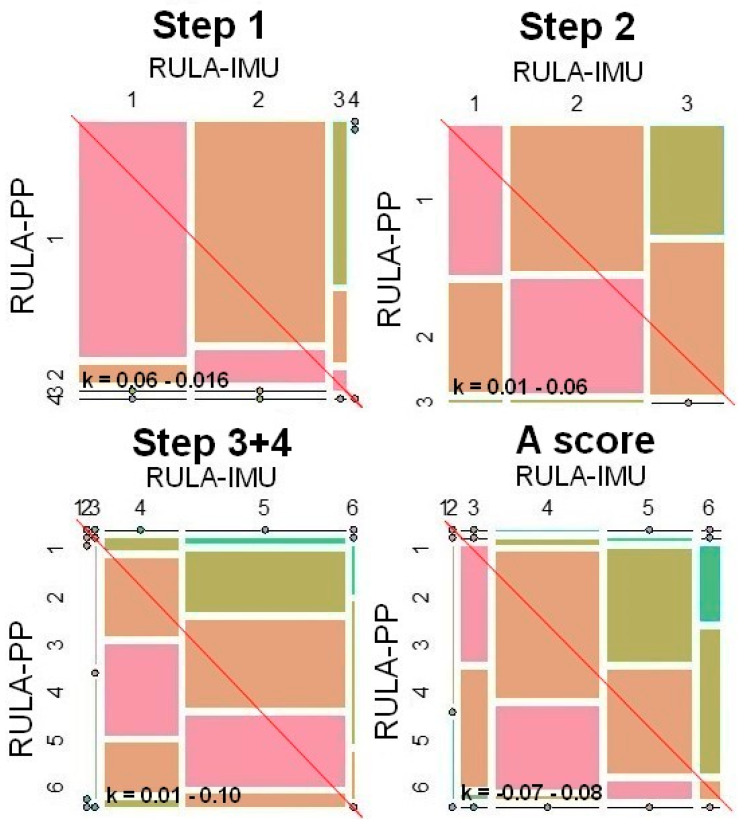
Mosaic plot of *area A—Arm and Wrist Analysis*. Red represents a difference of 0; orange represents a difference of 1; green–yellow represents a difference of 2; green represents a difference of 3; turquoise represents a difference of 4; blue represents a difference of 5; lilac represents a difference of 6; purple represents a difference of 7; and k is the kappa coefficient/weighted Cohen’s Kappa.

**Figure 3:**
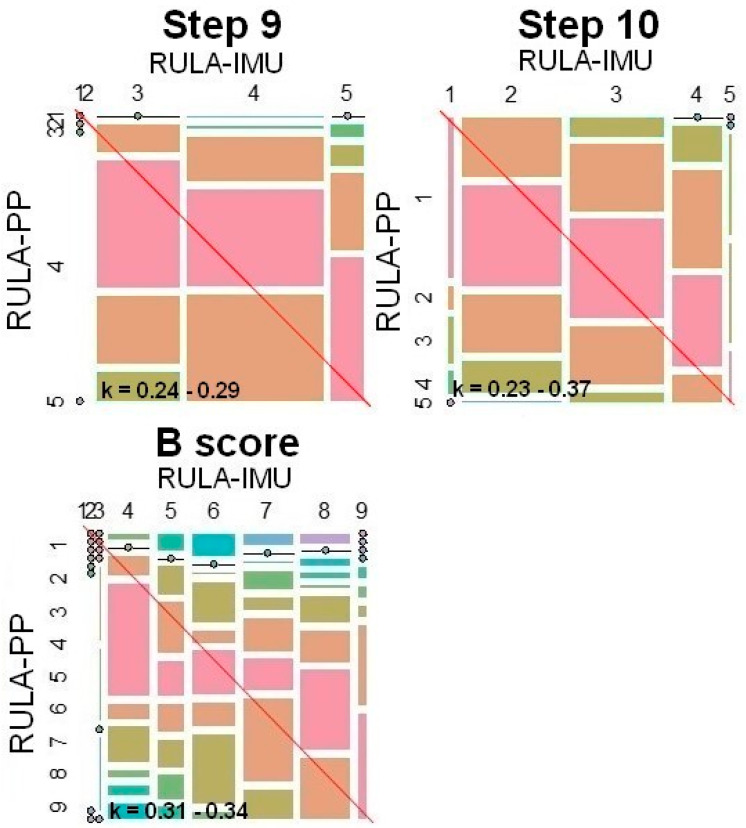
Mosaic plot of *area B—Neck, Trunk and Leg Analysis*. Red represents a difference of 0; orange represents a difference of 1; green–yellow represents a difference of 2; green represents a difference of 3; turquoise represents a difference of 4; blue represents a difference of 5; lilac represents a difference of 6; purple represents a difference of 7; and k is the kappa coefficient/weighted Cohen’s Kappa.

**Figure 4:**
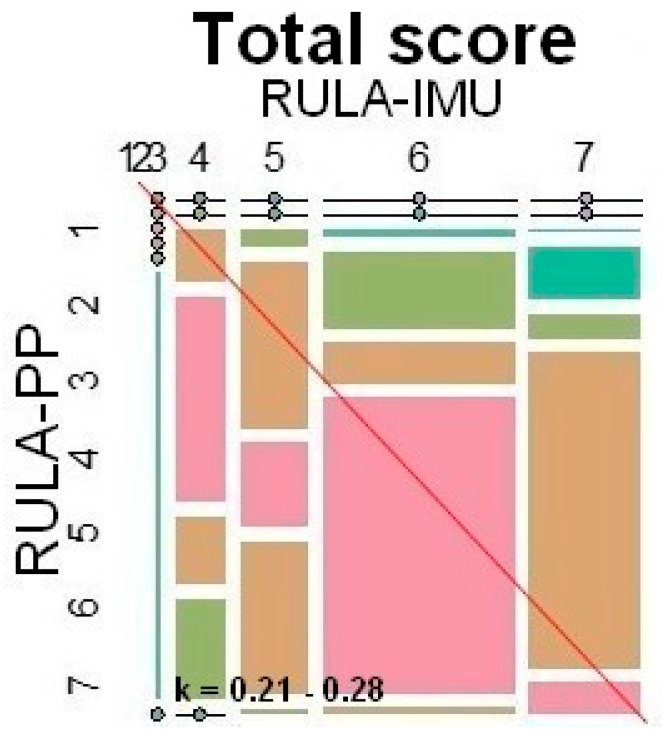
Mosaic plot of the *total score*. Red represents a difference of 0; orange represents a difference of 1; green represents a difference of 2; turquoise represents a difference of 3; lilac represents a difference of 4; and k is the kappa coefficient/weighted Cohen’s Kappa.

In the following, the *total score*, as well as *areas A* and *B* and their corresponding Cohen’s Kappa results, are presented as a mosaic plot, where the corresponding side presents 100% of the results in each case (Figure 2, Figure 3 and Figure 4).

As an example, the A Score and B score are discussed in more detail, since they correspond to the overall scores for *area A—Arm and Wrist Analysis*—and *area B*—*Neck, Trunk, and Leg Analysis*—and thus serve as the characteristic value of these areas. In addition, we take a closer look at the *total score*.

In Figure 2, for all three steps (1, 2 and 3 + 4) and the A score, almost the entire intersection is below the straight line, illustrating a higher point value of RULA-IMU.

At A score, the kappa coefficient according to Cohen is −0.07–0.08 (Table 1), the lowest agreement of the measurement methods of all the steps, as a summary of *area A*, and it reflects the lowest agreement of all. This lowest agreement of both methods can also be seen in the mosaic plot as, here, the largest quadrilaterals intersected by the diagonal are orange (=difference of 1). RULA-IMU has the tendency here for the point value to be one point higher than is the case with RULA-PP. This is because RULA-IMU has scores of between 3 and 6, with a weighting of mainly 4 and 5, whereas RULA-PP scored almost only 3 and 4.

In Table 1, the median of the method comparison in *area B* is 0. The associated weighted Cohen’s Kappa is higher in this range than in *area A*, but it is still between 0.25 and 0.39, which shows a weak agreement between the methods. Therefore, not only should the median be considered, but also all the data must be taken into account in their entirety in order to be able to assess the agreement as, otherwise, the median would lead to a false assumption. This relationship between the two methods is also evident in the mosaic plots of Figure 3, as, here, the red diagonal runs proportionately through more red boxes. However, the distribution pattern of the red areas does not show good method agreement, which is also clearly shown by the weighted Cohen’s Kappa. Accordingly, although there are similar proportions of red quadrilaterals below and above the red 45° diagonal running through each plot overall, due to the calculated median of 0, the location of the quadrilaterals and the point value allocation must be taken into account. If this fact is considered, the weighted Cohen’s kappa also shows in the mosaic plot that there is weak agreement between the methods because the associated value k is <0.4.

The B score has the second highest kappa coefficient, with k = 0.31–0.34, and, consequently, one of the highest agreements, which can also be further examined graphically in the mosaic plot (Figure 3). The mosaic plot shows that RULA-PP rates lower, whereas, for high RULA-IMU scores, RULA-PP rates raise for low RULA-IMU scores. Therefore, the agreement is rather low (even though one of the highest among all steps) despite a median difference of 0.

The mosaic plot shows a similar weight for RULA-IMU ratings between scores 6 to 8. RULA-PP on the other hand assigned most ratings to score 8.

In the *total score* (Figure 4), it can be seen that RULA-PP was awarded lower scores more often (=almost all red squares below the diagonal). However, the point value 6 was awarded the most by both methods (=largest red square). Above all, RULA-PP distributed the point value of 6 to 60% (40% of the scores are spread over other scores) and thus created a one-sided result.

## 4. Discussion

The aim of this study was to compare an already frequently used [28,29,30,31,32] and promising RULA application for the future by means of inertial motion analysis and integrated RULA analysis (RULA-IMU) with the conventional RULA application (RULA-PP). This comparison has not previously been made and is intended to provide a more detailed insight into possible differences in order to compare more effectively the results of both methods and place them in the scientific context.

In regard to the first question, *area A*—*Arm and Wrist Analysis* (step 1, 2, 3 + 4 and A score)—demonstrate a low agreement. RULA-IMU was frequently one assessment point above RULA-PP in the risk assessment with a low kappa coefficient, according to Cohen (k ≤ −0.07–0.16) (Table 1). This tendency was also clearly recognisable in the different patterns of the rectangles of the mosaic plots (Figure 2). According to this, the red 45° diagonal can separate these quadrangles into two parts (see methodology). In all steps of *area A—Arm and Wrist Analysis*—the squares of agreement was below the red diagonal and thus showed a bias towards higher values on the part of RULA-IMU.

This is possibly due to the fact that the individual steps of *area A* were more difficult for RULA-PP to grasp visually as they involved smaller angles and body parts that were hardly visible. This was especially evident for the wrist as its complex movements could only be assessed to a limited extent, particularly if there was an additional rotation in the forearm. This complicated the insight for the observer to assess all joint planes on a two-dimensional photo. In order to optimally determine the angle by means of a projection as a photo, the picture would have to be taken directly orthogonal to the joint to be assessed. However, this is not possible with several joints and with the several degrees of freedom that have to be assessed simultaneously. Since the projection generates a systematic error, the risk determined by RULA-PP appears lower than it might be.

To counteract this problem, the images were generated from two different perspectives (front top and rear side). In future analyses, a better insight would be possible if the photos were taken rather from above, in order to receive a better overview of the body positions to be judged in *area A—Arm and Wrist Analysis*.

In the experimental set-up, care was taken to create a real working environment, in that the instruments, the environment, and the working tools were similar to that of a practice. The phantom head used was not a real patient, and thus the study took place under laboratory conditions and not in a real workplace. In addition, it should be noted that the results are limited to static postures from dentistry and some of the participants were still students. Therefore, no general statements can be made for other work groups, but it can be assumed that the results are also applicable to them. To verify this, this study should be repeated in a real workplace with other working groups whose participants are professionals.

The step 3 + 4 for the assessment of the wrist to the neutral position from *area A* had the lowest kappa coefficient of all single steps with 0.01–0.10. In this step, the smallest joint of all joints observed by RULA was assessed. Here, the wrist was assessed in the sagittal, transverse, and frontal planes, which is difficult for the observer to detect, having only two available angles of view. In order to be able to assess the transversal and sagittal planes more precisely for RULA-PP, an additional camera should have been used here to record the test set-up from above.

Regarding the differences of the risk assessment in *area B—Neck, Trunk, and Leg Analysis* (research question 2)—the median of the differences was 0, after which the results of the risk assessment of both methods initially agreed. The B score (neck, trunk, leg score) had one of the highest agreements between RULA-IMU and RULA-PP, with a kappa coefficient of 0.31–0.34. Especially large body parts, such as the head and upper body, which do not perform any fine motor actions, were clearly visible to the observer in the photos. Consequently, these parts were easier to judge. However, this can only be seen as an apparent match since different risk assessments of the methods can be obtained from the B score (Figure 3). While the most frequently chosen score was 8 for RULA-PP, and the score for RULA-IMU was 7. The median of the differences in both methods seems to balance out the “outliers” at the ends of the scale again, which could result in a distortion of the statement. It is precisely these extreme differences that should prompt users of both systems to examine more critically their results and consider them in the consensus of the overall picture, which illustrate a supposed agreement between the measurement methods. Consequently, in *area B*, by balancing extreme values, it is suggested that both methods would be identical in this area.

The method differences of the individual RULA steps leading to the *total score* (research question 3) seem to be small (the median difference in the risk assessment of both methods was 0), but they do differ in the distribution of scoring values (Figure 4). RULA-IMU assigned a score of 7 much more often than RULA-PP. These two scores, however, lead to a diverging assessment of the risk of termination, i.e., whether measures should be taken soon or immediately. Therefore, they can have an impact in the practical implementation of ergonomic measures. The different evaluations of both methods become particularly clear with the result of k = 0.21–0.28 because these results stand for a weak agreement. This would be the case, among other things, with regard to the prevalence of the development of musculoskeletal disorders and associated potential consequences, such as the incapacity to work [33,34]. Although, in a broad overview, the measurement methods agree on the *total score*, the differences in their interpretation can have far-reaching consequences.

From these results, recommendations can be formulated for the targeted use of both methods. Under the premise that the kappa coefficient, according to Cohen (k), lies in the range of a weak agreement and thus below 0.39 for all results, both methods show barely any similarities (in their results). In this context, however, it must be taken into account that the originally developed RULA concept [9] was only conditionally applicable to the data collection of the IMUs sensors, and thus certain RULA steps for objectification were precisely defined [25]. Precisely, this accuracy is also an evident advantage of RULA-IMU. Reliable and reproducible values can be determined over the entire movement sequence (static and dynamic work activities), even under movements that are difficult to see and change frequently and quickly. Conversely, RULA-PP makes assessment more difficult because the human observer can only estimate angular dimensions. Consequently, the scores in this study also diverge.

As motion capture systems are increasingly used [12,13,14] to detect musculoskeletal diseases, for example, the inertial motion unit manufacturer Xsens has now caught up [35,36]. In future, it will be possible to upload data collected by IMU directly to the manufacturer and then receive a RULA evaluation. Since RULA-PP was used more frequently in older studies [37,38,39], it was not possible to compare the results with RULA-IMU until now. The trend to generate ergonomic analyses by IMUs will continue, and RULA-IMU is a good measure to obtain a risk analysis by RULA without the need to involve an observer.

One advantage of RULA-PP is its low-cost acquisition, in the sense of a trained observer. The optimal situation would be to have an assessor on site to continuously assess the risk over the duration of the entire process to be assessed. For an ideal assessment of the workflow and obtaining reliable data, a time-intensive installation of several cameras would be advantageous. However, this camera-based RULA-PP implementation only generates a still image, from which it is difficult to assess the angles of the individual extremities. Furthermore, the still image should always be taken from the same angle, preferably with several perspectives (front, back, top, side). However, RULA-PP may reveal its weaknesses in producing a rather low risk rating, even though it is often used and is a scientifically well-evaluated method. Here, RULA-PP scores one assessment point below RULA-IMU, especially in relation to the upper body posture (*area A*). RULA-PP assessments not only have a 26.83% error rate [6] but also systematically underestimate risk compared to RULA-IMU. In addition, RULA-PP has strong fluctuations in inter- and intra-rater reliabilities [40], which are not present in RULA-IMU, showing a good inter-rater reliability (0.61–0.99) and a good inter-rater reliability (0.61–0.99) of IMU [41]. It can, therefore, be concluded that additional expertise and experience in conducting risk assessments as an observer using the pen-and-paper method from a defined point of view cannot increase reliability decisively.

The statistical analysis by means of method comparison showed that RULA-IMU and RULA-PP rarely coincide. Furthermore, from utilising the applied statistics, it was possible to determine which methods tended to estimate the risk assessment higher or lower. Especially in *area A*, it should be taken into account that RULA-PP tends to estimate the risk to be lower. This is due to human limitations, such as the exact angle estimation with the human eye or difficulties in the detection of dynamic, as well as complex, movements. Here, it must be taken into account that it is precisely at the critical border between 6 (which still allows a wait-and-see attitude) and 7 (immediate intervention is necessary) that it must be verified exactly whether the score is really only 6 or whether immediate action is necessary [7] in the *total score*.

In addition, it is now possible to ensure the best possible and most accurate risk assessment in the workplace by providing recommendations for static postures. From now on, static postures can be compared by RULA-PP (whether old or new data) with RULA-IMU results. Now, it is known that the risk assessment of both methods has a systemic difference of 1, as RULA-IMU tends to give a higher risk.

In this study, we collected the data through static postures in order to ensure a high reliability on behalf of the observer. It is questionable whether the differences between the two measurement methods are the same for work that is more dynamic and complex. Therefore, in future studies of more dynamic movements at the workplace, it should be investigated whether the differences in RULA-IMU and RULA-PP observed in this study also apply to more dynamic situations.

## 5. Conclusions

The study shows that RULA-PP scores are, systematically, one point higher than RULA-IMU in assessing the risk of static motion in many areas.

These results can be very helpful for the classification of future ergonomic assessments (at least for static movements) using RULA-IMU in the RULA-PP-dominated scientific background of ergonomic analyses using RULA.

The study outcomes enable a comparison of RULA-PP results with those of RULA-IMU in predominantly static postures. This, in turn, helps to minimize risk assessment and the development of musculoskeletal diseases, as future risk assessments will increasingly be performed using IMUs.

## Figures and Tables

**Table 1:** Results of the statistical analysis of RULA-IMU and RULA-PP.

		Min. Kappa and Confidence Intervals	Max. Kappa and Confidence Intervals	Min. Median and IQR	Max. Median and IQR
Area A	Step 1	0.06 [−0.06–0.17]	0.16 [0.04–0.29]	0 (1)	0 (1)
	Step 2	0.01 [−0.04–0.09]	0.06 [0.03–0.17]	1 (1)	1 (1)
	Step 3 + 4	0.01 [−0.08–0.09]	0.10 [0.03–0.18]	1 (1)	1 (1)
	A Score	−0.07 [−0.16–0.02]	0.08 [0.01–0.15]	1 (1)	1 (2)
Area	Step 9	0.25 [0.14–0.36]	0.29 [0.17–0.41]	0 (1)	0 (1)
	Step 10	0.23 [0.11–0.35]	0.37 [0.26–0.49]	0 (1)	0 (2)
	B Score	0.31 [0.21–0.42]	0.34 [0.24–0.44]	0 (2)	0 (2)
	Final	0.21 [0.09–0.34]	0.28 [0.16–0.39]	0 (1)	1 (1)

## Data Availability

All data generated or analysed during this study are included in this published article.

## Abbreviations

ERATs	ergonomic risk assessment tools
RULA	Rapid Upper Limb Assessment
IMUs	inertial measurement units
RULA-PP	RULA paper and pen method
RULA-IMU	RULA kinematic data
D	dentist
DA	dental assistant
IQR	interquartile range
